# Reply to Szöllősi and Williams: The observation threshold mitigates the bias of transient genes on ancestral reconstruction

**DOI:** 10.1073/pnas.2619901123

**Published:** 2026-07-27

**Authors:** Vincent Gagnon, Miklós Csűrös

**Affiliations:** ^a^ Department of Computer Science and Operations Research, Université de Montréal, Montréal, QC H3T 1J4, Canada

We appreciate the thorough evaluation of our algorithms by Szöllősi and Williams (1). Their C-language implementation and the extended analysis of the 269-genome dataset provide valuable tests of reproducibility and replication. Notably, however, their analysis does not challenge our conclusion that large-scale reconciliations (2–4) hallucinate lateral gene transfers because of weak phylogenetic signal. The authors offer instead a case study in how uncorrected model fitting distorts deep ancestral reconstructions. They note that the unfiltered dataset leads to an absurdly “inflated” root genome, while filtering on minimum family size (4 to 10 genes) brings ancestral genome estimates into biologically plausible ranges at the expense of global model fit.

The apparent tension between the fitted model and the observed family-size distribution is amplified on the log–log plot in ref. 1, where families with at most 10 copies occupy almost half of the visual range. Such small families are formed mainly by transient genes, constituting an evolutionary “noise” characterized by high turnover and fleeting expansions. The experiments show that forcing a single homogeneous gain-loss-duplication (GLD) model to fit massive transient data (75% of families are singletons and 95% of families have 1 to 10 members) alongside core and shell families inevitably compromises the accuracy at deep nodes.

**Fig. 1.**
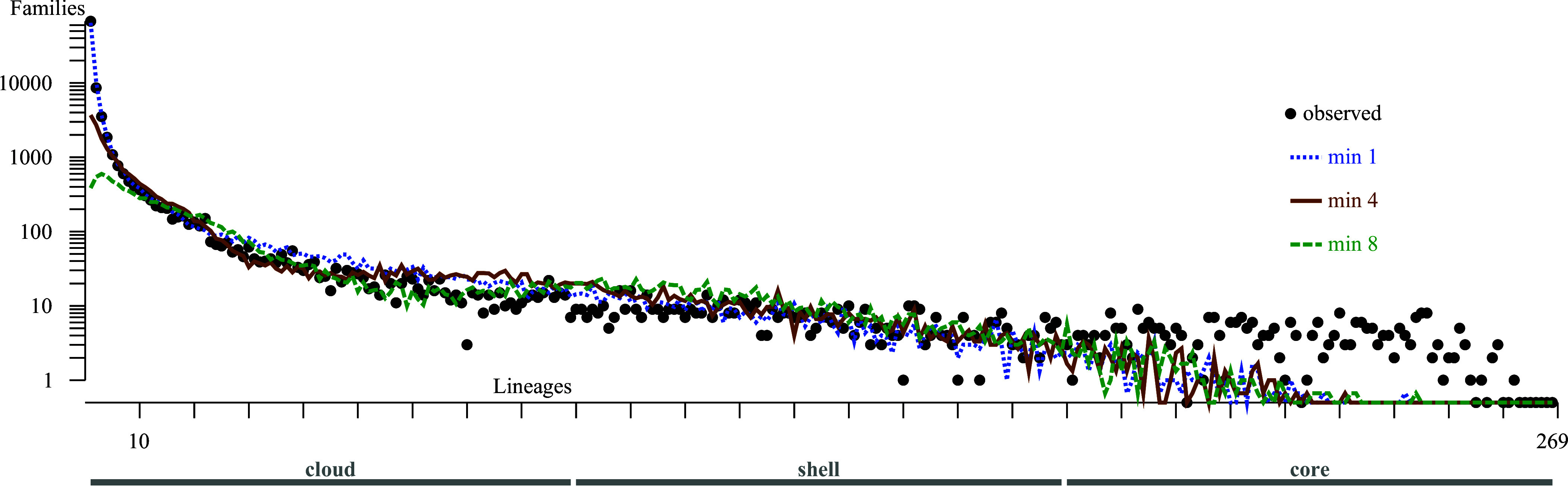
Distribution of family frequency (extant genomes with a copy) in optimized models (average in three simulations) with different sampling thresholds and all observed profiles; on log–linear scale. Simulated datasets have the same number of observed families (viz. at least 1, 4, or 8 copies) as the original.

Imposing a minimum size is not simply a data truncation exercise but a necessary safeguard against model misspecification. Fig. 1 shows the distribution of family frequency in models optimized with sampling thresholds Ω_min_ = 1, 4, 8 and the 269-genome dataset. Models with larger sampling bias filter out transient families and thus underestimate their frequency, but approximate more common families better. Unfortunately, too large thresholds also filter out valuable data for gain rates in smaller phylogenetic groups (in our dataset, multiple classes and phyla have four or fewer representatives) and terminal branches.

**Fig. 2.**
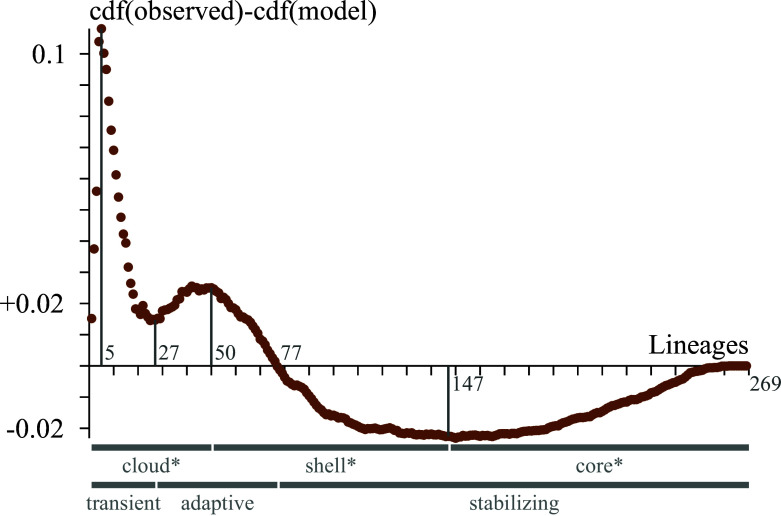
Cumulative residual plot for family frequency distribution (minimum four copies): difference between the cumulative distributions in the dataset (observation) and model simulations (average across three experiments); 1% difference amounts to ∼115 families. Data-dependent cloud, shell, and core regions (marked by asterisks) are separated by the last two local extrema. The first minimum and the zero-crossing define the transient, adaptive, and stabilizing force-regions. Transient influx and recurrent adaptive loss skew the distribution to the left with respect to the model; stabilizing conservation pushes families toward ubiquity.

Biologically interpretable phenomena that violate model assumptions either by dependent loss or by nonidentical rates emerge against the statistically rigorous background of the GLD model. Fig. 2 reveals that deviations between the GLD model and observed families are not simply random noise, but are primarily determined in three distinct regions by separate evolutionary forces: transient influx, adaptive loss, and stabilizing conservation (5). In order to advance beyond adjustable copy thresholds, we can aim to account for biological forces directly in the model architecture. Incorporating across-family rate variation or dependent family evolution promises a better segregation between stable, adaptive, and transient families; it is also possible to combine GLD inference for larger families with reconciliation methods for small families (where they can excel over copy-number inference).

Szöllősi and Williams highlight the exact pitfalls that our algorithm avoids while confirming its robustness on a scale beyond our original manuscript. In deep phylogenomics, forcing a global statistical fit over a heavily skewed distribution comes at the expense of biological reality.
